# Expression of cereblon protein assessed by immunohistochemicalstaining in myeloma cells is associated with superior response of thalidomide- and lenalidomide-based treatment, but not bortezomib-based treatment, in patients with multiple myeloma

**DOI:** 10.1007/s00277-014-2063-7

**Published:** 2014-04-01

**Authors:** Shang-Yi Huang, Chung-Wu Lin, Hsiu-Hsia Lin, Ming Yao, Jih-Luh Tang, Shang-Ju Wu, Yao-Chang Chen, Hsiao-Yun Lu, Hsin-An Hou, Chien-Yuan Chen, Wen-Chien Chou, Woei Tsay, Sheng-Je Chou, Hwei-Fang Tien

**Affiliations:** 1 Department of Internal Medicine, National Taiwan University Hospital, B4:0509, No.7, Chung-Shan South Road, 10002 Taipei, R.O.C Taiwan; 2 Department of Pathology, National Taiwan University Hospital, Taipei, Taiwan; 3 Department of Laboratory Medicine, National Taiwan University Hospital, Taipei, Taiwan

**Keywords:** Cereblon, Immunohistochemistry, Immunomodulatory drugs, Multiple myeloma, Prognosis

## Abstract

Cereblon (CRBN) is essential for the anti-myeloma (MM) activity of immunomodulatory drugs (IMiDs), such as thalidomide and lenalidomide. However, the clinical implications of CRBN in MM patients are unclear. Using immunohistochemical (IHC) staining on paraffin-embedded bone marrow sections, the expression of CRBN protein in myeloma cells (MCs) was assessed in 40 relapsed/refractory MM (RRMM) patients who received lenalidomide/dexamethasone (LD) and 45 and 22 newly diagnosed MM (NDMM) patients who received thalidomide/dexamethasone (TD) and melphalan/bortezomib/prednisolone (MVP), respectively. IHC staining were scored on a scale representing the diffuseness and intensity of positive-staining MCs (range, 0–8) and a score ≥4.5 was used for CRBN positivity (CRBN^+^) on a cut-point analysis of all possible scores and response of TD and LD. Compared to CRBN^+^ NDMM patients, CRBN^−^ NDMM patients had more international staging system (ISS) III (26 vs. 61 %, respectively; *P* = 0.006). In the LD and TD cohorts, the response rate (RR) was higher in CRBN^+^ patients than CRBN^−^ patients (LD 79 vs. 33 %, respectively; *P* = 0.005) (TD 75 vs. 29 %, respectively; *P =* 0.005); however, this trend was not observed in the MVP cohort. In the LD and TD cohorts, the positive and negative prediction value of CRBN^+^ for treatment response was 79 and 67 % and 75 and 71 %, respectively. Multivariate analysis showed that CRBN^+^ was a significant factor associated with superior RR for LD and TD. The data suggest that expression of CRBN protein in MCs assessed using the IHC is a feasible approach to predict the response of IMiDs in MM patients.

## Introduction

Multiple myeloma (MM) is a malignant plasma cell (PC) proliferation typically found in bone marrow (BM) [1]. Anti-MM treatment has advanced in the past decade with the availability of several novel agents that improve the survival rates of MM patients [1, 2]. These novel agents include immunomodulatory drugs (IMiDs), such as thalidomide and its derivatives, lenalidomide, and pomalidomide. Although several mechanisms have been proposed to explain the anti-MM effect of IMiDs [3], the precise molecular mechanisms remain unclear. Cereblon (CRBN) was recently identified as a primary target for thalidomide teratogenicity; thalidomide directly binds to CRBN and subsequently disrupts the function of CRBN-related E3-ubiquitin ligase complex (E_3_ULC), resulting in abnormal regulation of bone morphogenetic protease and fibroblast growth factors signaling pathways and of developmental programs that require their normal functions [4]. Furthermore, CRBN is required for the anti-MM activity of the IMiDs [5]. The absence and downregulation of CRBN expression in human myeloma cell lines result in IMiDs resistance, which is also supported by downregulation of CRBN expression at the time of lenalidomide resistance in MM patients [5].

CRBN was first identified by Higgins et al. [6] in patients with autosomal recessive nonsyndromic mental retardation. The human CRBN gene mapped at chromosome 3p26 contains 11 exons and is conserved from plants to humans. CRBN encodes a 442-amino acid protein with a molecular weight of approximately 51kD with an ATP-dependent Lon protease domain and several phosphorylation sites that selectively degrade short-lived polypeptides and regulate mitochondrial replication and transcription [7]. CRBN can interact with the DNA damage-binding protein-1 (DDB1), Cullin 4 (Cul4A or Cul4B), and regulator of Cullins 1 (RoC1) to form a functional E_3_ULC [8]. The CRBN-DDB1-Cul4A-RoC1 E_3_ULC (CRBN-based E_3_ULC) attaches polyubiquitin chains to target proteins for degradation through the ubiquitin–proteasome protein degradation pathway [7, 8]. Although the substrates of CRBN, as a putative substrate receptor of the CRBN-based E_3_ULC, remain unidentified, the CRBN-based E_3_ULC has auto-ubiquitination activity in the absence of their specific substrates, which is inhibited by thalidomide [4], suggesting that binding of thalidomide to CRBN may inhibit the function of CRBN-based E_3_ULC [7]. In addition to thalidomide, lenalidomide and pomalidomide also bind CRBN and inhibit the auto-ubiquitination of CRBN [9]. Very recently, two specific B-cell transcription factors, Ikaros family zinc finger-containing protein 1 (IKZF1; Ikaros) and 3 (IKZF3; Aiolos), were found to be the targets of lenalidomide-CRBN ubiquitination degradation in myeloma cells including not only cell lines but also primary myeloma samples [10, 11]. Binding lenalidomide or its analogues, thalidomide and pomalidomide, to CRBN would increase the binding of IKZF1 (Ikaros) and IKZF3 (Aiolos) proteins to the CRBN-based E_3_ULC, leading to increased ubiquitination and consequent degradation, which is toxic to myeloma cells [11]. Lenalidomide did not alter IKZF1 and IKZF3 mRNA levels, consistent with it acting posttranscriptionally [10]. Furthermore, under physiological conditions, IKZF1 and IKZF3 repress IL-2 gene expression in T cells but conversely stimulate expression of interferon-related factor 4 (IRF4) (a transcription factor essential for survival of myeloma cells) [11, 12]. In primary human T cells treated with lenalidomide, both IKZF1 and IKZF3 protein levels decreased markedly, suggesting that induction of IL-2 is mediated by derepression of the IL-2 gene expression by depletion of IKZF1 and IKZF3 [10, 11]. Thus, a decrease in IKZF1 and IKZF3 explains the perplexing question of how IMiDs can both activate the immune system (a boost in IL-2 production by T cells stimulates immune responses) and degrade B cell function (as the result of reduced IRF4 expression) simultaneously [12].

Several clinical studies have correlated the higher expression of CRBN gene in myeloma cells with the superior treatment response of a lenalidomide-based regimen [13] and a pomalidomide-based regimen [14], as well as longer progression-free survival during thalidomide maintenance therapy [15]. However, the requirement for high-quality clinical samples, such as myeloma cells enrichment by cell sorting, limits the validation of such quantified transcriptional expression of the CRBN gene method to every MM patient. In addition, the lack of a consensus protocol to amplify the CRBN gene is a crucial problem [16]. In particular, CRBN frequently undergoes mRNA alternative splicing, and several isoforms have been described in MM [16, 17]. Some of the identified mRNA isoforms were even not translated [17]. The arbitrarily used cutoff level for the transcriptional level of CRBN gene expression is also difficult to determine consistently [14, 15]. Therefore, the design of the quantitative reverse transcription PCR (qRT-PCR) test to assess CRBN expression levels is critical and can yield variable results [16]. Low CRBN expression levels were detectable in CD138 negative cells, representing non-myeloma cells within a BM microenvironment [13], which may result in a misinterpretation of the CRBN gene expression level if the sorting of CD138 positive myeloma cells is not sufficiently pure. Therefore, immunohistochemical (IHC) staining may be an alternative approach for differentiating the myeloma cells and non-myeloma cells components in BM, and is easier to use for MM patients, including those with a low percentage of plasmacytosis in BM. Because the clinical implication of CRBN translational protein expression, rather than transcriptional gene expression, in MM patients treated with IMiDs has not been efficiently examined, we retrospectively analyzed the expression of CRBN protein in myeloma cells using IHC staining on paraffin-embedded BM tissues in MM patients who had received thalidomide or lenalidomide plus dexamethasone treatment to correlate the clinical features of MM patients with CRBN protein expression and evaluate the possibility of CRBN protein as a biomarker to predict the treatment response of IMiDs.

## Methods

### Patients and bone marrow samples

Since January 2011, a total of 40 patients with relapsed and/or refractory MM (RRMM), who had uniformly received lenalidomide and dexamethasone (LD) as their salvage treatment, were enrolled. BM biopsies of these RRMM patients were collected around the commencement of LD (median of −2 days, ranging from −3 to 2 days). Two additional cohorts of patients with newly diagnosed MM (NDMM) were enrolled; one cohort had thalidomide and dexamethasone (TD, *N* = 45) and the other cohort had melphalan, bortezomib, and prednisolone (MVP, *N* = 22) as their induction regimens. The BM samples of the NDMM patients at diagnosis were also obtained. The treatment schedules for LD [18], TD [19], and MVP [20], as well as the related dosage adjustment [1] were described previously. This study was approved by our institutional ethics committee, and written informed consent was obtained from all patients in accordance with the Declaration of Helsinki.

### Treatment response

The treatment response, progression-free survival (PFS), time to progression (TTP), duration of response (DOR), and overall survival (OS) were evaluated according to the IMWG consensus criteria [21, 22].

### Immunohistochemistry staining

The procedures of immunohistochemistry (IHC) staining in our laboratory were described as previously [23] with optimization for this study. Briefly, the BM biopsied samples were fixed in 10 % neutral buffered formaldehyde for at least 24 h, decalcified with Shandon TBO-2 decalcifier (Thermo Scientific, US) for 2 h, and embedded in paraffin. Paraffin-embedded BM tissue sections measuring 4 to 5 μm were deparaffinized in xylene, rehydrated with ethanol, and rinsed in PBS. After deparaffinization and rehydration, the slides were placed in the target retrieval solution (S1700, Dako, Denmark) and heated (90 °C to 99 °C) for 40 min. Endogenous peroxidase was then blocked with 3 % hydrogen peroxide (Dako, Denmark); blocking nonspecific protein binding with 10 % ovalbumin (EndoGrade®, Hyglos GmbH, Germany). After blocking, the slides were incubated with the primary antibody at room temperature for 30 min. The primary antibodies were monoclonal mouse anti-human CD138 (IgG_1_, MI15, Dako, Denmark) at a dilution of 1:100, and polyclonal rabbit anti-human cereblon antibody (IgG, 11435-1-AP, Proteintech, US) at a dilution of 1:50. After incubation with universal biotinylated link antibody and peroxidase-conjugated streptavidin, the reaction was achieved with the DAB substrate-chromogen solution using Universal Dako LSAB® + Kit (K0679, Dako, Denmark) according to the instructions of the manufacturer, followed by counterstaining with hematoxylin (00–8001, Invitrogen Camarillo, CA) for 10 min. Hepatic tissue obtained from the institutional tissue bank was used as a positive control for CRBN IHC staining, because of its high expression of CRBN [24]. Cardiac tissues were used as a negative control because of its no expression of CRBN [24]. Monoclonal rabbit anti-human IgG_1–4_ antibody (EPR4421, Abcam Inc, MA, US) at a dilution of 1:500 was used as a negative idiotype control. Each batch of IHC slides was accompanied by the positive and negative control. The location of myeloma cells were identified by CD138 positive membrane staining. CRBN immunostained slides were scored as previously described [25] with modification. In brief, each slide of BM trephine core biopsy immunostained for CD138 was firstly visualized at ×100 magnification to determine three “hot areas”, namely areas containing the maximum number of CD138 positive myeloma cells. The three “hot areas” were then identified at another slide immunostained with anti-CRBN antibody by using the very near slice to the CD138 immunostained slice and were examined at ×400 magnification. A brown granular or diffuse cytoplasmic and/or nuclear staining for CRBN within myeloma cells were considered to be positive. Then, a diffuseness score was assigned, which represented the estimated diffuseness of CRBN positive cells within the hot area (0, none; 1, <1⁄100; 2, 1⁄100 to <1⁄10; 3, 1⁄10 to <1⁄3; 4, 1⁄3 to 2⁄3; and 5, >2⁄3). Next, an intensity score was assigned, which represented the average intensity of CRBN positive cells (0, none; 1, weak, 2, intermediate; and 3, strong). The diffuseness and intensity scores were then added to obtain a total score, which ranged from 0 to 8. The total score in each hot area was obtained and the average total score for the three hot areas were calculated and presented. The CRBN IHC staining was interpreted by two independent and trained reviewers, and the scores were assigned. The results of equivocal cases were interpreted and determined by a third independent reviewer.

### Statistics

Chi-square or Fisher’s exact tests were used for between-group comparisons of the discrete variables. A two-sample *t* test or one-way ANOVA was used for between-group comparison of the means. Pearson’s correlation tests were used to analyze the continuous variables, and Spearman correlation was used for the nominal variables. Kaplan–Meier survival curves were constructed to estimate PFS, TTP, DOR, and OS, and the differences between groups were compared using the log-rank test. Inter-reviewer agreement was evaluated using Cohen’s kappa value [26]. In the analyses, identified salient variables for clinical and laboratory data were categorized as described previously [27]. The variables were as follows: age ≥60 years, stage ≥international staging system (ISS) III, light chain isotype, BM plasmacytosis ≥30 %, beta-2-microglobulin (β_2_M) ≥2.5 mg/L, hemoglobin (HB) ≥10g/dL, white blood cell ≥4.0×10^9^/L, platelet ≥1.5×10^11^/L, lactate dehydrogenase (LDH) ≥upper normal limit (UNL), alkaline phosphatase ≥UNL, calcium (Ca) ≥2.4 μmol/L, creatinine (Cr) ≥2 mg/dL, and C-reactive protein (CRP) <UNL (0.8 mg/dL). Factors that provided statistically significant predictive power in univariate analysis were further tested using multivariate regression analysis of the linear, logistic, or Cox type, with forward stepwise selection. All directional *P* values were two-tailed, with a *P* value of 0.05 or less considered significant for all tests. All analyses were performed using SPSS 19.0 software (Chicago, IL, USA).

## Results

### Patients

The salient clinical characteristics of the 40 RRMM patients at commencement of LD are shown in Table 1. The median therapy prior to LD was 2 lines (range of 1–5 lines). The median time from diagnosis of MM to LD treatment was 34 months (range of 3–151 months). In total, 93, 75, and 40 % of patients had been exposed to thalidomide, bortezomib, and high-dose chemotherapy followed by autologous stem cell transplantation (HDT/AuSCT), respectively. The salient features of NDMM patients at diagnosis in two additional cohorts are shown in Table 1.

**Table 1:** Salient characteristics of the RRMM patients who had LD treatment and the NDMM patients who had TD or MVP as their induction treatment

Disease	RRMM	NDMM	
Treatment	LD	TD	MVP
Pt number	(*n* = 40)	(*n* = 45)	(*n* = 22)
Sex (M/F)	25/15	28/17	11/11
Age (yrs)^a^	62.8 ± 9.6	60.5 ± 12.2	60.3 ± 11.3
ISS, *n* (%)			
I/II	28 (70)	30 (67)	9 (41)
III	12 (30)	15 (33)	13 (59)
Isotype, *n* (%)			
IgG	27 (67)	20 (44)	15 (68)
IgA	9 (23)	15 (33)	6 (27)
IgD	0 (0)	2 (4)	0(0)
Light-chain	4 (10)	8 (19)	1 (5)
Kappa: lambda	1:1	1.3:1	1.2:1
HB (gm/dL)^a^	11.4 ± 2.2	9.8 ± 2.8	8.9 ± 2.3
WBC (×10^9^/L)^a^	5.7 ± 2.6	6.4 ± 2.8	5.6 ± 3.8
PLA (×10^9^/L)^a^	1.7 ± 1.0	1.9 ± 0.8	1.7 ± 0.9
Creatinine (mg/dL)^a^	1.1 ± 0.9	1.9 ± 2.1	1.6 ± 1.1
Calcium (μmol/L)^a^	2.2 ± 0.2	2.2 ± 0.3	2.3 ± 0.4
LDH (IU/L)^a^	431 ± 310	394 ± 153	605 ± 1062
ALP (IU/L)^a^	200 ± 137	228 ± 214	152 ± 72
CRP (mg/dL)^a^	1.9 ± 4.8	2.0 ± 2.7	1.6 ± 3.4
Alb (gm/dL)^a^	3.8 ± 0.5	3.6 ± 0.9	3.5 ± 0.8
β_2_M (mg/L)^a^	6.7 ± 12.2	8.6 ± 13.7	10.9 ± 8.7
Plasma cell in BM (%)^a^	48.6 ± 32.4	56.9 ± 29.8	67.3 ± 28.0
Cytogenetic abnormalities, *n* (%)	8 (20)	8 (18)	2 (9)
EMM, *n* (%)	7 (18)	7 (16)	6 (27)

*Alb* albumin, *ALP* alkaline phosphatase, *BM* bone marrow, *CRP* C-reactive protein, *EMM* extramedullary myeloma, *F* female, *HB* hemoglobin, *ISS* international staging system, *LDH* lactate dehydrogenase, *M* male, *NDMM* newly diagnosed multiple myeloma, *PLA* platelet, *Pt* patient, *RRMM* relapsed and/or refractory multiple myeloma, *WBC* white blood cell, *β*_*2*_ *M* beta_2_-microglobulin

^a^Mean ± SD

### CRBN immunohistochemical staining and associated clinical features

**Fig 1:**
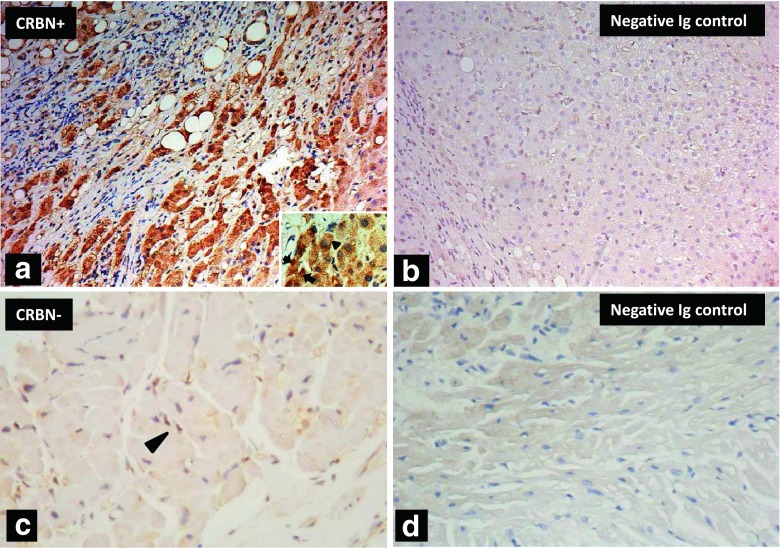
Positive and negative control for the CRBN immunohistochemical staining. Positive control for CRBN IHC staining in hepatocytes **(a)** (×200) and a higher magnification (×400) for the cellular details with positive granular cytoplasmic staining (*arrow head*) and positive nuclear staining (*arrow*) is inserted. Negative anti-idiotype (Ig) control showing no IHC signals in the same hepatic tissues as in **a** by using anti-idiotype antibody **(b)** (×200). Negative control for CRBN IHC staining in cardiac tissues is shown in **(c)** (×400) and a nonspecific nuclear staining is noted in some myocardial cells (*arrow head*). Negative anti-idiotype (Ig) control in the same cardiac tissue as in **c** by using anti-idiotype antibody is shown in **(d)** (×400)

**Fig 2:**
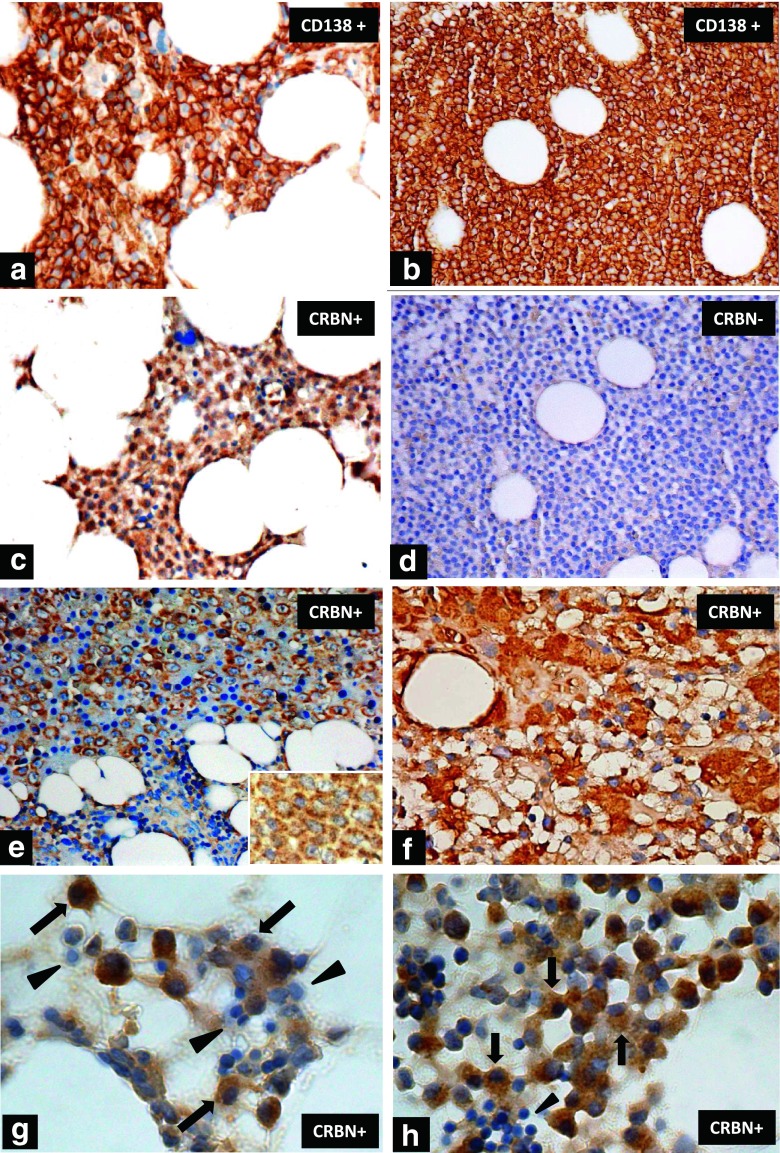
The immunohistochemical staining of CRBN in myeloma cells. Aggregated myeloma cells highlighted by CD138 membranous staining in **(a)** and **(b)** (×400). Positive CRBN cytoplasmic/nuclear staining in myeloma cells with the same slice as in **a** is shown in **(c)** (×400). Negative CRBN staining in myeloma cells with the same slice as in **b** is shown in **(d)** (×400). Another positive CRBN cytoplasmic staining in immunoblastic-like myeloma cells **(e)** (×400) and a higher magnification (×1000, oil lens) for the cellular details of granular cytoplasmic pattern is inserted. Aggregates of myeloma cells with intense cytoplasmic staining for CRBN and less distinct nuclei **(f)** (×1000, oil lens). Typical myeloma cells stained positive for CRBN (*arrow*) were shown in **(g)** and **(h)** (×1000, oil lens). Several CRBN negative myeloid and mononuclear cells were noted in **g** (*arrow head*) and a cluster of CRBN negative erythrocytes are noted in **h** (*arrow head*)

The positive and negative controls of CRBN IHC staining on hepatic and cardiac tissues are shown in Fig 1. The typical myeloma cells stained positive for CRBN is shown in Fig 2c, e–h. By contrast, the myeloma cells stained negative for CRBN is shown in Fig 2d. On the basis of a univariate cut-point analysis of all possible scores and treatment response in patients who had received LD and TD therapy, an average IHC total score ≥4.5 was used to define CRBN positivity (CRBN^+^), since which generated the most balanced positive and negative predictive value (PV^+^ and PV^−^, respectively) for the treatment response in the LD and TD cohorts (Table 2). There was a strong and positive correlation between the average diffuseness score and intensity score (Pearson’s correlation in reviewer A and B was 0.810 and 0.904, respectively; both *P* < 0.001). There was a good correlation for the average total score between the reviewers (coefficient of Pearson’s correlation, 0.891; *P* < 0.001). The inter-reviewer agreement on the CRBN^+^ showed a moderate to high correlation with Cohen’s kappa value of 0.702 (*P* < 0.001) and Spearman’s correlation coefficient of 0.706 (*P* < 0.001). In the LD cohort, the myeloma cells of 19 (48 %) of the 40 patients were CRBN^+^. Compared to the CRBN^+^ RRMM patients, CRBN-negative (CRBN^−^) RRMM patients had significantly higher WBC (*P* = 0.022). Among the 67 NDMM patients, the myeloma cells of 39 (58 %) patients were CRBN^+^. Compared to the CRBN^+^ NDMM patients, CRBN^−^ NDMM patients had significantly more ISS III (26 vs. 61 %, respectively; *P* = 0.006), higher level of calcium (2.2 vs. 2.4 μmol/L, respectively; *P* = 0.045), and lower platelet counts (2.0×10^11^/L vs. 1.5×10^11^/L, respectively; *P* = 0.037). A significantly negative correlation was observed between CRBN IHC status (positive vs. negative) and β_2_M (*r* = −0.283; *P* = 0.022) in NDMM patients.

**Table 2:** The response rate, positive and negative predictive value according to the different cutoff of the average total score for CRBN IHC in LD and TD cohort

LD cohort (*n* = 40)						TD cohort (*n* = 45)				
IHC score cutoff	CRBN^+^ (*n*); RR (%)	CIRBN^−^ (*n*); RR (%)	*P* value	PV^+^ (%)	PV^−^ (%)	CRBN^+^ (*n*); RR (%)	CRBN^−^ (in); RR (%)	*P* value	PV^+^ (%)	PV^−^ (%)
>3	22; 68.2	18; 38.9	0.110	68.2	61.1	35; 60.0	10; 50.0	0.720	60.0	50.0
≥4	20; 75.0	20; 35.0	0.025	75.0	65.0	33; 63.6	12; 41.7	0.306	63.6	58.3
>4	20; 75.0	20; 35.0	0.025	75.0	65.0	31; 67.7	14; 35.7	0.057	67.7	64.3
≥4.5	19; 78.9	21; 33.3	0.005	78.9	66.7	28; 75.0	17; 29.4	0.005	75.0	70.6
>5	17; 82.4	23; 34.8	0.004	82.4	65.2	25; 76.0	20; 35.0	0.008	76.0	65.0
>5.5	15; 86.7	25; 36.0	0.003	86.7	64.0	20; 70.0	25; 48.0	0.224	70.0	52.0
>6	12; 83.3	28; 42.9	0.035	83.3	57.1	17; 70.6	28; 50.0	0.222	70.6	50.0

*LD* lenalidomide/dexamethasone, *TD* thalidomide/dexamethasone, *RR* response rate (≥partial response), *PV*+ positive predictive value, *PV*- negative predictive value

### Expression of CRBN was associated with better treatment response in IMiDs-based treatment (not MVP treatment)

The median treatment cycle for LD was 11 cycles (range of 1–33 cycles). The median treatment duration for the TD and MVP cohorts was 7 months (range of 1–38 months) and 10 months (range of 1–13 months), respectively. In the LD and TD cohorts, CRBN^+^ patients had a significantly superior treatment response (overall response rate, terms of partial response or better) to compared CRBN^−^ patients (LD cohort 79 vs. 33 %, respectively; *P =* 0.005) (TD cohort CRBN^+^ vs. CRBN^−^, 75 vs. 29 %, respectively; *P =* 0.005); however, this did not occur in the MVP cohort (CRBN^+^ vs. CRBN^−^, 91 vs. 82 %, respectively; *P =* 1.000). The details of the treatment response to various regimens between CRBN^+^ and CRBN^−^ patients are shown in Table 3. In the LD and TD cohorts, the PV^+^ and PV^−^ of CRBN^+^ for treatment response was 79 and 67 and 75 and 71 %, respectively. From commencement of LD, after a median follow-up of 28 months, no significant difference was observed between CRBN^+^ and CRBN^−^ patients regarding the PFS (median, 8 vs. 8 months, respectively; *P* = 0.7439), TTP (median, 9 vs. 10 months, respectively; *P* = 0.9177), DOR (median, 9.5 months vs. not reached yet, respectively; *P* = 0.4752), and OS (median, not reached yet vs. 27 months, respectively; *P* = 0.2279). In the TD cohort, 31 of the 45 NDMM patients did not undergo further HDC/AuSCT, and among these 31 NDMM patients, no significant difference was observed between CRBN^+^ and CRBN^−^ patients regarding the PFS (median, 15 vs. 7 months, respectively; *P* = 0.4600), TTP (median, 27 vs. 7 months, respectively; *P* = 0.6179), and DOR (median, 12 vs. 6 months, respectively; *P* = 0.7217).

**Table 3:** The treatment response (according to IMWG consensus criteria) of MM patients to various regimens according to CRBN IHC staining status

Treatment	LD			TD			MVP		
CRBN status (*n*)	Positive (19)	Negative (21)	*P* value	Positive (28)	Negative (17)	*P* value	Positive (11)	Negative (11)	*P* value
	*n* (%)			*n* (%)			*n* (%)		
CR^a^	0 (0)	1 (5)	0.032	2 (7)	0 (0)	0.022	3 (27)	1 (9)	0.550
VGPR	4(21)	1 (5)		7 (25)	0 (0)		2 (18)	4 (36)	
PR	11 (58)	5 (23)		12 (43)	5 (29)		5 (46)	4 (36)	
SD	3 (16)	10 (48)		6 (21)	9 (53)		1 (9)	2 (18)	
PD	1 (5)	4 (19)		1 (4)	3 (18)		0 (0)	0 (0)	
ORR (≥PR)	15 (79)	7 (33)	0.005	21 (75)	5 (29)	0.005	10 (91)	9 (81)	1.000

^a^Denote immunonegative complete response

*CR* complete response, *VGPR* very good partial response, *PR* partial response, *SD* stable disease, *PD* progressive disease, *ORR* overall response rate

### Expression of CRBN is an independent factor associated with treatment response of IMiDs

In the LD cohort, the salient characteristics that significantly correlated with treatment response were CRBN^+^, HB ≥10g/dL and CRP < UNL. Further multivariate analysis showed that CRBN^+^ (*r* = 7.409; 95 % confidence interval (CI) 1.57–34.933; *P* = 0.011) and HB ≥10g/dL (*r* = 6.236; 95 % CI 1.127–34.498; *P* = 0.036) were independent factors correlated with treatment response. In the TD cohort, CRBN^+^, ISS I/II, non light-chain type, and Cr <2 mg/dL were associated with the treatment response; however, only CRBN^+^ (*r* = 6.034; 95 % CI 1.328–27.422; *P* = 0.020) and ISS I/II (*r* = 8.807; 95 % CI 1.704–45.520; *P* = 0.009) remained independent factors after multivariate analysis (Table 4).

**Table 4:** Univariate and multivariate analyses among salient features and CRBN status between patients who had response to the TD induction treatment and those who did not

	With response (*n*=26)	Without response (*n* = 19)	*P* value	Univariate analysis	Multivariate analysis
Item	*n* (%)			Odds ratio (95% CI)	
CRBN^+^	21 (81)	7 (37)	0.005	7.200 (1.868–27.749)^a^	6.034 (1.328–27.422)^a^
ISS I/II	23 (88)	8 (42)	0.001	10.542 (2.331–47.669)^a^	8.807 (1.704–45.520)^a^
Non light-chain type	25 (96)	12 (63)	0.006	14.583 (1.607–132.334)^a^	–
Cytogenetic abnormalities	5 (19)	3 (16)	1.000		
PC in BM >=30%	17 (65)	12 (63)	1.000		
HB ≥10g/dL	16 (62)	6 (32)	0.071		
PLA ≥1.5×10^11/uL	21 (59)	11 (33)	0.111		
ALB ≥3.5g/dL	14 (54)	12 (63)	0.541		
Cr <2.0mg/dL	25 (96)	12 (63)	0.013	12.500 (1.349–115.795)^a^	–

*ALB*, albumin; *BM*, bone marrow; *CI*, confidence interval; *Cr*, creatinine; *HB*, hemoglobin; *ISS*, international staging system; *PC*, plasma cell; *PLA*, platelet; *WBC*, white blood cell

^a^Statistical significance

## Discussion

To our knowledge, this is the first study to show that CRBN protein expression assessed by IHC in myeloma cells of BM paraffin-embedded tissues is associated with superior treatment response to LD in RRMM patients and TD in NDMM patients. Similar to CRBN gene expression studies [14, 15], our data indicate that CRBN is a crucial factor for the anti-MM effect of IMiDs. A recent study also showed that higher CRBN protein expression, determined by the overall pixel intensity of fluorescence targeting the CRBN protein within CD138^+^ cells in BM samples, correlated with superior treatment response to LD than lower CRBN protein expression [28]. Similar to other studies [15, 28], such expression of CRBN protein was not associated with treatment response in the regimen without IMiDs (e.g., MVP), suggesting that CRBN is a unique biomarker for predicting the response of IMiDs in MM patients. Although a significant difference on time to event in this study was not reached because of limited patient numbers, a trend in favor of CRBN^+^ NDMM patients compared to CRBN^−^ NDMM patients was noted regarding longer PFS, TTP, and DOR. These findings suggest that identifying CRBN protein expression by the IHC may be a clinically feasible approach for predicting treatment response and outcome of IMiDs in MM patients.

Unlike the qRT-PCR and gene expression profiling (GEP), which results in dose-dependent association between the CRBN gene expression level and treatment response [14, 15], IHC staining produces, in general, non-quantified results and is reviewer-dependent. In this study, by using the IHC scores consisted of both diffuseness and intensity of CRBN expression within myeloma cells, the semi-quantified results could be obtained. This immunostain score was adopted from the scoring system verified ever in breast cancer [25], and such scoring systems have been introduced in order to overcome variations, particularly for markers that are used for making therapeutic selections [29]. Unlike the homogenous pattern of cancer cells in solid tumors, myeloma cells within BM always aggregated separately [1]. Therefore, we initially picked up three hot areas of mostly aggregated myeloma cells and evaluated the diffuseness and intensity scores of CRBN within these sampled hot areas. Cutoff levels for assessing whether a tissue is “positive” or “negative” can vary for the same antigen. The optimal cutoff level, herein, was chosen by the best balanced PV^+^ and PV^−^ for the treatment response of LD and TD. Notably, the optimal cutoff level chosen in the LD (RRMM) cohort was nearly the same as that seen in the TD (NDMM) cohort (Table 2), suggesting that this method and the cutoff level was generally reproducible. Automated image analysis might be one of the alternative ways to minimize the subjective bias on IHC interpretations between different reviewers, however, which is time- and cost-consuming and is still predominantly a research tool [29]. Another problem for the automated image analysis systems is that such analysis assesses the amount of staining by measuring absorption, so the non-linear relationship that occurs at higher and/or lower levels between amount of antigen and intensity can result in inaccurate readings [29]. By using the immunostain score for CRBN and an optimal cutoff level of average total score ≥4.5 for determining CRBN^+^ in the well-trained reviewers, there was a Cohen’s kappa score of 0.702 for the inter-reviewer agreement, suggesting a fair to good agreement beyond chance [26]. Unfortunately, no data on the predictivity relating CRBN gene expression and treatment response of IMiDs with published qRT-PCR methods could be compared with ours. Nonetheless, IHC method may offer additional advantages over molecular methods (e.g., GEP), such as no requirement for cell purification by sorting, can be still used in patients with quite low percentage of myeloma cells in BM, lower expense and routinely available in most of the laboratories, ease of use, and faster turnaround time, and is still able to offer a cost-effectiveness predictive value for the response in several cancer treatments [25, 29]; so, it might be an alternative method, other than qRT-PCR or GEP, to be applied in the future study designed for biomarker enriched cohort. Moreover, morphology is preserved in the IHC procedure, allowing for recognition of immunostain heterogeneity and confirmation that the identified positivity is localized to which subcellular compartments. In CRBN^+^ myeloma cells, there were mostly both positive cytoplasmic and nuclear staining (like that seen in Figs 2c, f), but some were only positive cytoplasmic staining (Fig 2e). It was reported that the subcellular localization of CRBN was primarily in the juxtanuclear area and cytoplasm, but inconsistently in nucleus [4, 24, 30]. The clinical significance of the different subcellular localization of CRBN is not clear yet.

In this study, several clinical salient features were correlated with CRBN protein expression status. In general, compared to CRBN^+^ patients, CRBN^−^ patients had a more advanced disease status, such as more prevalent ISS III. Prior studies have indicated that MM patients with lower CRBN gene expression had more ISS III [5, 15], which is consistent with our results. In addition, higher levels of CRBN expression indicate a low risk of disease [14]. A correlation was observed between CRBN expression and chromosomal hyperdiploidy, especially Trisomy 3 [14, 16], which is a prognostic factor toward improved survival rates in MM patients [1, 27]. However, after adjustment of other clinical prognostic factors using multivariate analysis, the CRBN^+^ in this study remained an independent factor associated with the treatment response of LD and TD (Table 4).

The treatment response to LD in RRMM and TD in NDMM patients was similar to those in other studies [31]. However, 21 % RRMM and 25 % NDMM patients did not respond to the LD and TD regimen, respectively, despite expression of CRBN protein within the myeloma cells, suggesting the possibility of mechanisms of resistance that do not involve CRBN. A prior study demonstrated that inducible activation of the Wnt/β-catenin pathway by lenalidomide treatment mediated lenalidomide resistance in MM [32], which affects downstream targets, such as CCND1 and MYC. Alternatively, in addition to CRBN, approximately 30 proteins were identified as potential substrate receptors [called DDB1-CUL4-associated factors (DCAFs)] contributing to ubiquitination of cellular proteins [7]. Competition occurs between CRBN and other DCAFs for binding to DDB1 [4]. Therefore, when IMiDs bind to CRBN, various DCAFs may bind to DDB1 and exhibit differing cellular functions, including resistance. Further studies to prove this hypothesis are required. Recently, a novel truncating mutation and R283K point mutation of CRBN were observed in an extramedullary plasmacytoma from a MM patient with clinical resistance to lenalidomide, which is, however, a rare event (4 %) in MM patients [33]. Acquired deletion of the CRBN gene was observed in an in vitro study [5], as well as CRBN gene copy reduction [9]. However, further examination of CRBN status on additional human myeloma cell lines (HMCLs) and MM patients suggested that copy number abnormalities affecting the CRBN gene were rare events in MM [5]. In addition, alternating splicing transcripts and modification of translational proteins of CRBN must be further examined. Exon 10 of CRBN, which contains a portion of the IMiD-binding domain, is not present in one (CRBN-002) of the isoforms of CRBN reported. The functional consequence of CRBN-002 is unclear; however, it may be a marker of drug resistance [17].

CBRN can be a biomarker for IMiDs sensitivity; however, it is unclear whether it can be targeted through pharmacological means to induce chemosensitization and reverse resistance. The molecular mechanism to regulate the expression of CRBN is unclear. A single NF-E2-related factor 2 (Nrf2)/antioxidant response element (ARE) (Nrf2/ARE) site in the upstream promoter region of mouse CRBN is responsible for most hypoxia/reoxygenation (H/R)-dependent increases in CRBN expression [34]. In addition, the overexpression of Nrf2 or treatment with an Nrf2 pathway-activating chemical compound (e.g., tert-butylhydroquinone) can induce the expression of the endogenous CRBN gene [34]. These results suggest that CRBN gene expression can be controlled by reactive oxygen species (ROS)-dependent signaling. Therefore, it is interesting to determine whether the expression of CRBN in myeloma cells is also regulated by ROS signaling, and whether the activation of the Nrf2 pathway can upregulate CRBN expression in CRBN^−^ myeloma cells to recapture the sensitivity of IMiDs.

This study has other limitations. First, because of the retrospective nature of this study, the treatment dosage and length varied among individual patients, which resulted in bias in the response and outcome assessment. Further prospective study with uniformly IMiDs-based treatment protocol and more patients enrolled is required. Second, up to date, all the seven commercialized anti-CRBN antibodies, including the one used in this study, are all polyclonal. The IHC staining of CRBN may be enhanced by the further optimizing IHC protocol and also by the novel in-house developed monoclonal anti-CRBN antibody [17]. Finally, no risk-associated fluorescent in situ hybridization (FISH) data were available in this study; notably, the updated reports on the correlation between CRBN expression and FISH-defined high-risk or standard-risk MM showed controversial results [14, 35].

## Conclusion

Positive CRBN protein expression assessed using IHC was associated with superior treatment outcomes in MM patients who received LD or TD regimens; our data indicate that CRBN protein can be a biomarker to predict the treatment response of IMiDs.
